# Predicting Child Development Across Literacy, Physical, Learning, and Social‐Emotional Domains Using Supervised Machine Learning: A Cross‐Sectional Study Based on MICS 2019 Bangladesh

**DOI:** 10.1002/hsr2.71434

**Published:** 2025-11-04

**Authors:** Faizul Islam, Golam Morshed Suhel, Mahmud Afroz, Md Aminul I. Apu, Md Jewel Rana, Tofajjel Hossain, Zaibunnesa Ziba, Md Fahim Shariar, Mohammad Nayeem Hasan

**Affiliations:** ^1^ Department of Statistics Shahjalal University of Science & Technology Sylhet Bangladesh; ^2^ Department of Sociology University at Albany, State University of New York USA; ^3^ Black Hills Energy Fayetteville Arkansas USA; ^4^ Department of Public Health, School of Applied Sciences The University of Mississippi, University Mississippi USA; ^5^ Department of International Relations Bangladesh University of Professionals Dhaka Bangladesh; ^6^ Department of Computer Science Independent University Dhaka Bangladesh

**Keywords:** algorithms, cognition, early childhood development, machine learning, psychomotor performance, social behavior, statistical models

## Abstract

**Background and Aims:**

Early childhood development (ECD) plays a vital role in shaping a child's health and well‐being, influenced by child, family, and environmental factors. To prevent long‐term impairments, early detection and intervention are crucial. Using MICS 2019 data, this study applies supervised machine learning to predict ECD across four key domains and identify the most significant predictors and economic strategies.

**Methods:**

In this study, using data of 9346 children obtained from Multiple Indicator Cluster Surveys (MICS) 2019, we evaluated and compared five classifiers: CART, Random Forest, XGBoost, Logistic Regression, and Support Vector Machines (SVM). We have addressed four early developmental domains as our target variables: literacy, numeracy, physics, learning, and social‐emotional development of children. Five‐fold cross‐validation was used to ensure appropriate test error rate estimations and reduce bias. To handle the data imbalance, the Synthetic Minority Oversampling Technique (SMOTE) is used.

**Results:**

The analysis shows that most children are developing normally in the learning (90.58%) and physical (98.70%) domains, while delays are highest in literacy‐numeracy (71.37%) and social‐emotional (27.57%) domains. Among the machine learning models evaluated, Random Forest consistently performed best across all domains, achieving the highest accuracy, particularly in learning (0.83) and physical (0.97) domains. Feature importance analysis identified maternal education, child age, regional location (Division), and socioeconomic status (Wealth Index) as key predictors. Early childhood education and books read at home also play important roles in cognitive and learning outcomes, guiding targeted interventions for child development.

**Conclusions:**

The results show notable differences in early childhood development, particularly in social‐emotional and literacy‐numeracy domains. Socioeconomic status, early learning experiences, and parental education are key predictors, while physical and social‐emotional development are influenced by resources, regional factors, and nutrition. These findings can guide targeted interventions and policies for holistic child development.

**Abbreviations**
AUC‐ROC: Area Under The Receiver Operating Characteristic Curve
CART: Classification and Regression Trees
ECD: early childhood development
ECDI: early childhood development index
LMICs: low‐ and middle‐income countries
MICS: Multiple Indicator Cluster Survey
SMOTE: Synthetic Minority Oversampling Technique
SVM: Support Vector Machine
VIF: variance inflation factor

## 1 Background

Early childhood development is crucial for lifelong health and well‐being, offering both significant growth opportunities and vulnerability to harm [1]. Key aspects of child development, such as self‐regulation, early relationships, knowledge acquisition, and skill development, are shaped by neurobiology, caregiver interactions, and the caregiving environment, influencing a child's learning readiness and future health outcomes [2, 3, 4]. The first one thousand days of life are critical for physical, social, emotional, and cognitive development. Experiences during this time frame have a significant impact on a child's future development [5].

Poor birth outcomes, inadequate stimulation, malnutrition, chronic health concerns, organic problems, psychological and familial variables, and other environmental effects can all cause developmental delays [6]. Early detection and intervention are crucial to prevent long‐term disability [7]. This underscores the importance of early detection to initiate immediate steps with family involvement, seeking to avoid delays, encourage emerging abilities, and offering a more engaging and protected environment [6].

The exact prevalence of developmental delay is uncertain; however, the World Health Organization (WHO) estimates that approximately 10% of the population in each country lives with some form of disability [8]. Worldwide, approximately 52.9 million children under the age of five face developmental disabilities, with a significant majority (95%) living in low‐ and middle‐income nations (LMICs). In the United States, around 15% of children, and in England, 2.17% of children under the age of five, experience developmental issues [7, 9]. However, it is currently projected that 250 million children in LMICs are not developing to their full potential [10]. Data from 63 LMICs collected between 2010 and 2016 indicated that 25.3% of children showed developmental deficits, with regional rates varying from 10.1% in Europe and Central Asia to 41.4% in West and Central Africa [11]. In Bangladesh, The Multiple Indicator Cluster Survey (MICS) estimated that 30% of children were not developmentally on track in 2012, decreasing to 25.3% in 2019 based on the Early Childhood Development Index (ECDI) [12].

Many factors, both biological and environmental, impact children's development; some of these elements protect and improve it, while others have a detrimental effect [6]. Children in poverty face higher developmental delays than those from wealthier backgrounds due to increased exposure to risks and long‐term effects on brain development from poverty and trauma [13, 14]. Inadequate nutrition and poor maternal nutritional status during pregnancy can lead to intrauterine growth restriction, adversely affecting brain development and highlighting the crucial role of maternal nutrition in fetal survival, growth, and development [15]. Studies have discovered a connection between childhood malnutrition and some variables, including a person's socioeconomic status, demographics, household and environmental factors, parental characteristics, child‐feeding practices, child morbidity, vitamin intake and vaccination coverage, residence, and geographic location [16]. Early childhood programs play a vital role in aiding the cognitive and physical growth of young children, with those involved demonstrating notably greater developmental advancement than their peers [13].

Conventional statistical methods, which are based on restrictive assumptions, may struggle to model this complexity and hinder research into risk prediction tools [17]. However, machine learning, leveraging advances in data from birth cohorts, health records, imaging, and other sources, offers new potential for modeling these interactions and identifying optimal predictive patterns for early interventions [18]. Traditional statistical methods focus on inferring relationships based on predefined assumptions, while machine learning (ML) offers improved prediction and reveals complex patterns that traditional methods may miss [19]. Machine Learning (ML) is used to make predictions by learning patterns from data without relying on predefined assumptions or rules [20, 21]. ML models are non‐parametric, excelling at identifying significant variables for prediction [22, 23]. A primary benefit of machine learning is its capacity to manage numerous highly interactive predictors and nonlinear relationships, which can enhance predictive accuracy as data quantity and diversity increase [24].

Machine learning does not always guarantee better predictive performance than traditional methods. Effectiveness relies on the outcome, the quantity and nature of features, and the interactions between features. It's important to trial and report traditional methods like logistic and linear regression before considering more complex ML models [25]. This study introduces a machine learning approach using multilabel classifiers to analyze childhood developmental data and compares cost‐effective prediction models for developmental delays using a large MICS survey sample. Using information from the MICS survey, this study attempts to apply and assess supervised machine learning models for forecasting early childhood development in four key domains: learning, physical, literacy‐numeracy, and social‐emotional. Finding the best and most economical predictive models while emphasizing the important variables affecting developmental outcomes is the goal.

To achieve this goal, our research employs supervised machine learning techniques that surpass conventional statistical models. This study aims to create a more precise and comprehensible framework for comprehending the multidimensional aspects of Early Childhood Development (ECD) by pinpointing and prioritizing essential predictors across developmental domains. The importance of this study is that it could identify risk and protective factors that are specific to each domain. This would give policymakers and practitioners useful information that they could use to create targeted interventions that improve the well‐being of children. To reach these goals, we use feature importance analysis to find and rank the most important predictors across developmental domains. This method also captures complex, nonlinear relationships. The models are put through a lot of tests with different evaluation metrics to make sure they are strong and reliable. We perform individual‐level modeling for each developmental domain instead of forming a singular aggregate ECD index, enabling us to discern distinct patterns and risk factors that inform domain‐specific interventions.

This study fills an important gap in the current ECD literature. Previous studies have predominantly utilized regression‐based frameworks, frequently presenting results in the consolidated ECD index. These methods often hide vulnerabilities that are specific to a certain area and make it harder to understand, which makes it harder to come up with targeted policy responses. Conversely, our study offers a more sophisticated and pragmatic comprehension of child development through the incorporation of domain‐level machine learning models, feature importance analysis, extensive evaluation metrics, and individual‐level domain‐specific modeling. To our knowledge, no previous research has utilized this integrated approach, rendering our study both innovative and socially significant.

## 2 Materials and Methods

### 2.1 Data Source

UNICEF conducts MICS, an extensive, multifaceted, nationally representative household survey. Standardized questionnaires are used in this study to collect data and important information about children. This survey primarily emphasizes the health of reproductive women, interventions for child health, nutrition for children, and early childhood development. A comparable set of socioeconomic data about people and families is also gathered by MICS [12].

### 2.2 Study Design

**Figure 1:**
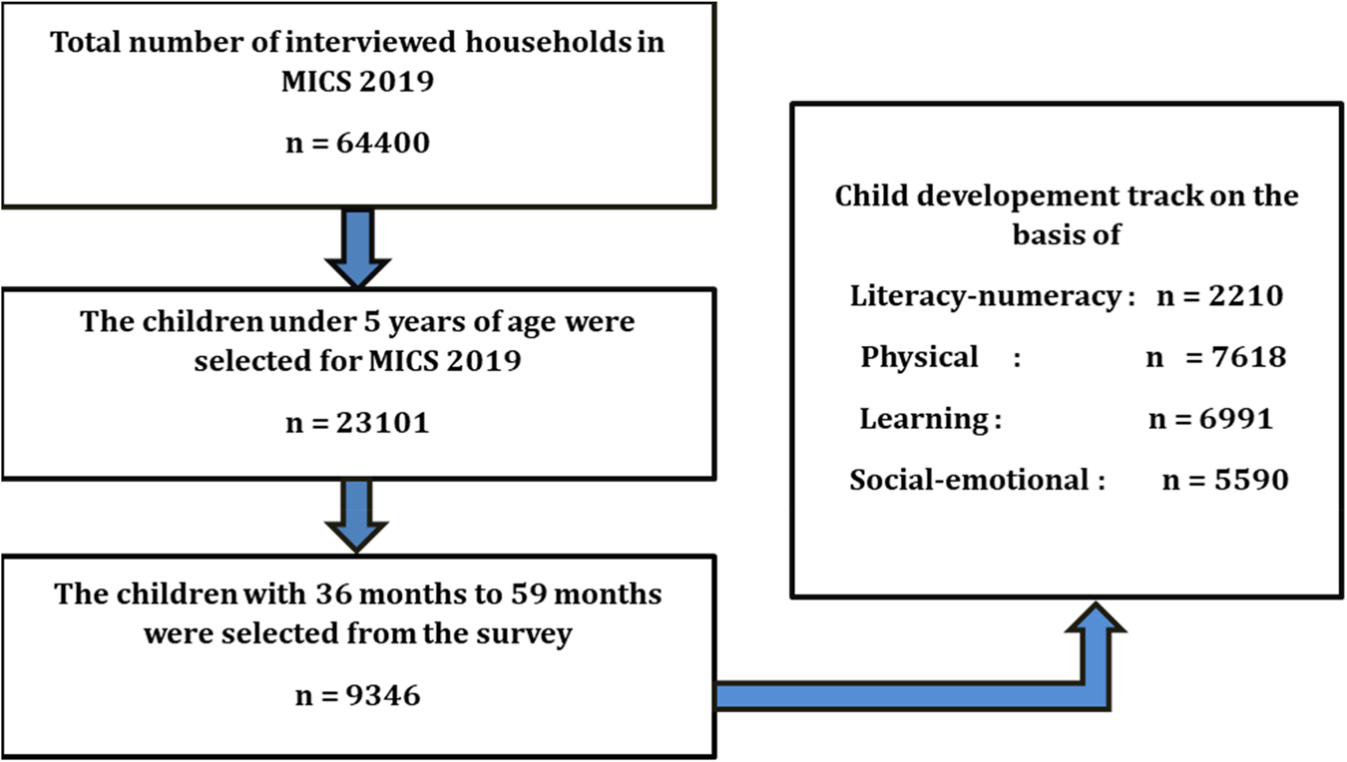
Schematic diagram of the sample for the analytical study.

Using a two‐stage cluster sampling procedure, the MICS survey selects randomly from households with children under 5 years. The 2019 Bangladesh MICS survey collected data from 64,400 households. Among them, a sample of 61,246 households with a response rate of 60,878 (99.4%) was used in the 2019 MICS survey. Regarding women's and children's health, MICS provides a comprehensive picture of Bangladesh's eight divisions (Dhaka, Chittagong, Sylhet, Rajshahi, Rangpur, Barisal, Khulna, Mymensingh). Districts were identified as the primary sample strata for sample selection at stage two. An age range of 36 to 59 months was selected for the 9346 children included in this study in 2019 (Figure 1). Primary sampling units (PSUs) were utilized to establish the census enumeration areas. These PSUs were selected from each sample area employing a systematic PPS (probability proportional to size) sampling approach, utilizing the anticipated sizes of the enumeration regions according to the latest population census [12].

### 2.3 Study Variables

We have taken four early developmental domains‐child literacy‐numeracy development, child physical development, child learning development, and child social‐emotional development as outcome variables. Each outcome variable is dichotomous. To determine the potential factors linked to the status of childhood developmental domains, independent features included the age and sex of the child, the child's place of residence (rural vs. urban), division, the mothers' educational level (secondary complete or higher, secondary incomplete, primary complete, and primary incomplete), the wealth index (richest, middle, and poorest), the religion (Islam and others), the sex and ethnicity of the household head (Bengali and others), the mother's age, early childhood diseases, nutritional status (underweight, stunting, wasting, and overweight), the early childhood educational program, books, toys, and sanitation facilities (improved and improved). For every item the mother/caregiver answered “yes,” we assigned each child a score of 1, and for every item she answered “no,” we assigned a score of 0.

### 2.4 Data Processing and Handling Imbalanced Data

To deal with missing values, we cleaned the data before developing the model. To preserve data quality and avoid bias during model training, records with missing entries were excluded from the analysis. Since the proportion of missing data was relatively small, deletion was preferred over imputation to ensure that only complete cases were included in the modeling process. The data used in this study didn't have any outliers or inconsistencies. To maintain the distribution of outcome classes, the data set was then split into training (80%) and testing (20%) subsets. Within an imbalance pipeline that simply uses SMOTE on the training folds, we have used GridSearchCV (5‐fold). Hyperparameters included criterion, max_depth, min_samples_split, min_samples_leaf, and max_features for CART/Random Forest; n_estimators, learning_rate, max_depth, min_child_weight, subsample, and colsample_bytree for XGBoost; kernel and regularization parameters for SVM. The final models were evaluated on the held‐out test set after being refitted on the entire training set and chosen based on mean CV performance.

We have used five‐fold cross‐validation. The most widely utilized values of k are ten (10) or five (5) because it is thought that these two numbers provide estimates of test error rates that are neither too biased nor too variably high [26]. Five sets are randomly selected from the data set; each set has nearly the same number of characteristics as the data set. The learning method is applied to the top four sets after each segment is held out in turn. The accuracy is then calculated using the holdout set. This procedure will reduce any potential bias brought about by the holdout approach, which reserves a specific amount for training and utilizes the remainder for testing [27].

Data imbalance was a problem we encountered in our case. In two domains—physical and learning development—the ratios between majority and minority classes are 98.7:1.3 and 90.6:9.4, respectively, indicating a severe imbalance. In contrast, the ratios between literacy and numeracy and social and emotional development, on the other hand, are 71.37:28.63 and 72.43:27.57, indicating a moderate imbalance. An imbalanced data set is any set of data that has an uneven split among its classes [28]. Chawla et al. introduced an innovative approach termed Synthetic Minority Oversampling Technique (SMOTE), which creates the “synthetic” sample instead of replacing the instances of the minority class [29]. To deal with class imbalance, we used SMOTE in this study. Model validation procedures were carried out to keep attention on this risk, even if SMOTE successfully enhances classification balance. In some situations, it may introduce synthetic noise or overfitting.

The variance inflation factor (VIF) was used to test multicollinearity; a cutoff value of 5 was chosen because a VIF value greater than 5 denotes significant multicollinearity [30]. We used the VIF of threshold 5 to remove explanatory variables to address multicollinearity. The transparency and clarity guidelines suggested the “Guidelines for reporting of statistics for clinical research in urology” by Assel et al. [31] for appropriate statistical reporting in clinical research are adhered to in the reporting of techniques and analytical procedures in this investigation [31].

### 2.5 Analysis

#### 2.5.1 Classifiers

In machine learning, various classifiers are employed to predict categorical outcomes based on input features. In this study, we explore the effectiveness of several machine learning algorithms to classify early childhood developmental outcomes. The models tested include Decision Trees, Random Forest, XGBoost, Support Vector Machines (SVM), and Logistic Regression, each with distinct advantages for handling classification tasks.

*Decision Tree:* In many diverse domains, including pattern recognition, image processing, and machine learning, decision trees are an effective and popular strategy [32]. Decision trees, which emerged as a flowchart‐like structure, are especially useful for classification and regression problems since they model decisions and their potential outcomes. The components of a typical tree include roots, branches, and leaves. Decision Tree adheres to the same format. It is made up of leaf nodes, branches, and root nodes [33].

*Random Forest:* Random Forest is a very widely used supervised machine learning algorithm that is used both in classification and regression tasks. Bootstrap Aggregation, also known as bagging, is an ensemble machine learning algorithm. The random forest consists of many decision trees. Randomization is incorporated into the construction process, which involves choosing sample and feature subsets, guaranteeing the independence of each decision tree, enhancing classification accuracy, and boosting generalization capability [34, 35].

*XGBoost:* XGBoost, or Extreme Gradient Boosting, is a powerful and modified gradient boosting algorithm that stresses speed and performance. To improve the precision and resilience of predictive models, it makes use of sophisticated optimization strategies, including parallel processing and regularization. In contrast to the traditional GBDT, which optimizes using first‐order derivative information, XGBoost has significantly improved optimization. To accelerate the model's convergence, it uses the second‐order Taylor expansion of the loss function, reserving the information of the first‐order derivative and adding the information of the second‐order derivative [36].

*Support Vector Machines (SVMs):* SVMs are a strong and adaptable supervised machine learning technique. The core concept of SVM is identifying the ideal hyperplane to separate the data points of multiple classes in the feature space. When a linear separation of the data is not achievable, SVM uses kernel functions to map the data into a higher‐dimensional space [37]. SVM attempts to achieve good generalization performance on unseen data by optimizing the margin between the closest data points of the various classes (referred to as support vectors).

*Logistic Regression:* A fundamental statistical method that is typically applied to binary classification issues in machine learning is logistic regression. Logistic regression converts its output using the logistic sigmoid function to deliver a probability value that may subsequently be translated to two or more discrete classes. Logistic Regression has three categories: Binary logistic regression, Multinomial logistic regression, and Ordinal logistic regression [38].

#### 2.5.2 Performance Metrics

We have used a total of six evaluation metrics here—Accuracy, precision, recall, F1‐score, Area Under the Receiver Operating Characteristic Curve (AUC‐ROC) score, and specificity. The primary method for evaluating errors in classification problems is the confusion matrix. They consist of all details related to misclassifications, such as the count of misclassified items for each original class pair in which items are meant to be classified, and the incorrect class where items are wrongly classified [39]. Here, TP, TN, FP, and FN represent true positives, true negatives, false positives, and false negatives, respectively.

$$\mathrm{Accuracy}=\frac{\text{TP}+\text{TN}}{\text{TP}+\text{TN}+\text{FP}+\text{FN}}$$

$$\mathrm{Precision}=\frac{\text{TP}}{\text{TP}+\text{FP}}$$

$$\mathrm{Recall}=\frac{\text{TP}}{\text{TP}+\text{FN}}$$

$$F1-\mathrm{Score}=\frac{2\times (\text{Precision}\times \text{Recall})}{\text{Precision}+\text{Recall}}$$

$$\mathrm{Specificity}=\frac{\text{TN}}{\text{TN}+\text{FP}}$$

The AUC‐ROC was used to evaluate the model's overall discriminative capacity across varying classification thresholds.

#### 2.5.3 Feature Importance Analysis

In machine learning, understanding the relative importance of features (predictor variables) is crucial for identifying the most influential factors affecting the outcome variable. Feature importance refers to the contribution of each feature in improving the predictive performance of a model. Various methods for calculating feature importance exist, with the most common being those based on decision trees, ensemble methods, and gradient boosting techniques [40, 41]. For this study, we performed a feature importance analysis using the best‐performing model identified in our previous experiments. This model was selected based on its superior performance in classifying early childhood development outcomes, evaluated using metrics such as accuracy, precision, recall, and F1‐score. Since the preserved predictors showed sufficient independence and interpretability for policy‐oriented analysis, no further feature selection or dimensionality reduction methods (such as PCA or RFE) were employed.

#### 2.5.4 Software Used

All analyses were conducted using Python (version 3.12.11), with scikit‐learn (version 1.6.1) for machine learning model implementation and evaluation. Seaborn (version 0.13.2) and Matplotlib (version 3.10.0) were used for data visualization, including feature importance and performance metrics. For numerical computation, NumPy (version 2.0.2) was used. Model validation and cross‐validation were performed using scikit‐learn to ensure robust results.

#### 2.5.5 Ethical Approval

Our study was entirely based on an analysis of publicly available health survey datasets from the [12], which are freely accessible online after all personally identifiable information has been deleted. UNICEF and the Bangladesh Bureau of Statistics (BBS) examined and approved the MICS procedures. During the interview process, participants gave their informed consent. This study did not need the ethical approval of the relevant institution because it involved the analysis of secondary data.

## 3 Results

**Figure 2:**
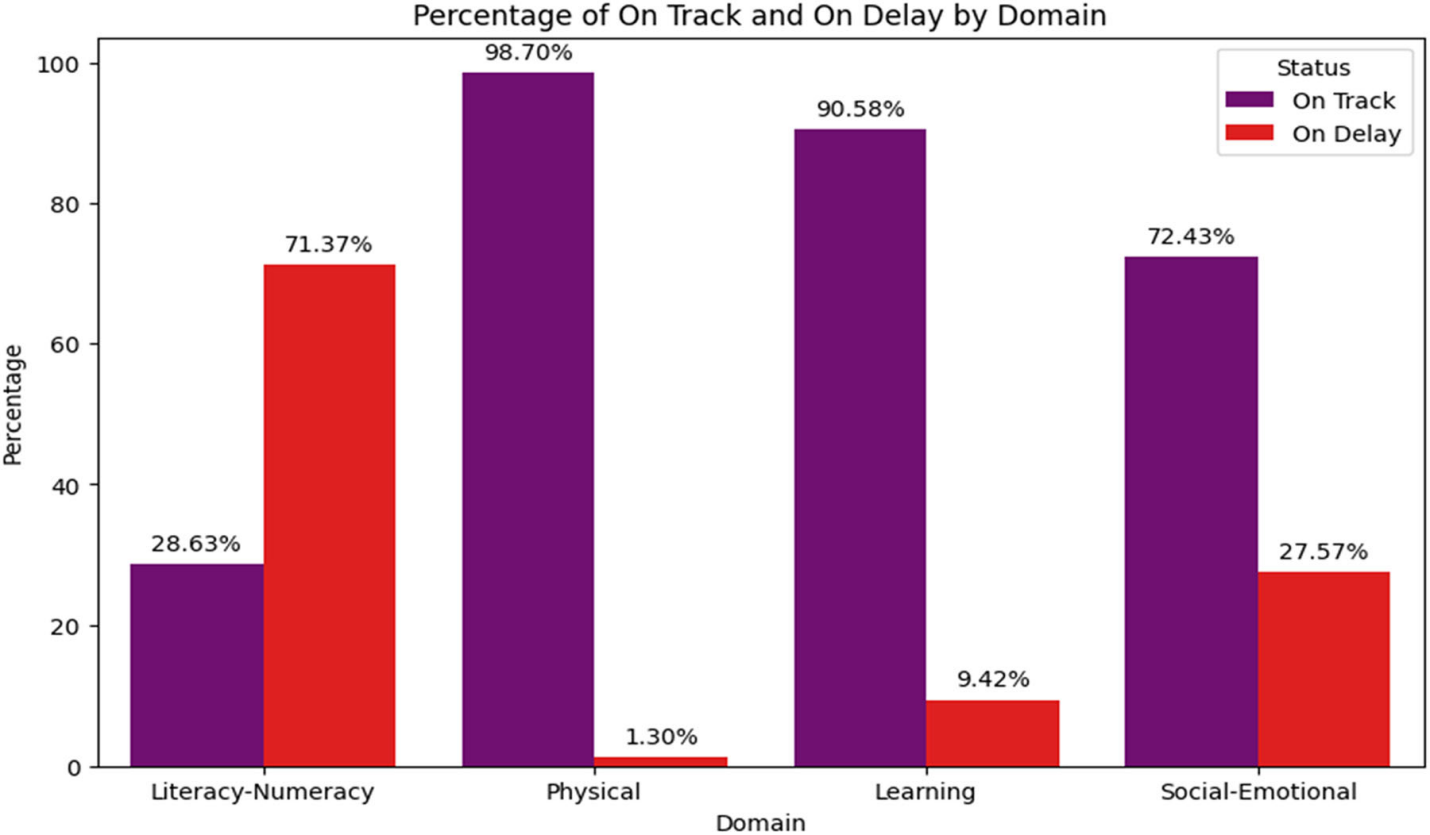
Distribution of children's developmental outcomes across all domains.

Most children are On Track in Physical (98.70%) and Learning (90.58%) domains, while Social‐Emotional development shows 27.57% On Delay. The Literacy‐Numeracy domain has the highest percentage of children On Delay (71.37%), highlighting significant developmental challenges in this area (Figure 2).

MICS 2019 [12] data show a nearly equal gender distribution among children under the age of five, with 51.48% men and 48.52% women. According to the data, 81.54% of children live in rural areas, with 18.46% living in cities. In the domain of literacy and numeracy, 27.33% of males are on track, while 30.01% of females are on track, indicating a slightly higher percentage in female literacy and numeracy development. In terms of physical development, the percentages for males and females are nearly identical, at 98.72% and 98.69%, respectively, on track. In the learning development domain, 91.30% of female children are on track, compared to 89.91% of males. In terms of social‐emotional development, 68.97% of males are on track, while girls are on track at a higher percentage (74.24%). This reflects the fact that a higher percentage of females than males are reaching the social‐emotional benchmarks (Table 1).

**Table 1:** Distribution of early childhood developmental status across all domains.

Characteristics	Literacy‐numeracy		Physical		Learning		Social‐emotional	
	Developmentally on track		Developmentally on track		Developmentally on track		Developmentally on track	
	Yes	No	Yes	No	Yes	No	Yes	No
	*N* (%)	*N* (%)	*N* (%)	*N* (%)	*N* (%)	*N* (%)	*N* (%)	*N* (%)
Household head's sex								
Male	1878 (28.55)	4701 (71.45)	6495 (98.72)	84 (1.28)	5971 (90.76)	608 (9.24)	4759 (72.34)	1820 (27.66)
Female	332 (29.15)	807 (70.85)	1123 (98.60)	16 (1.40)	1020 (89.55)	119 (10.45)	831 (72.96)	308 (27.04)
Ethnicity of household head								
Bengali	2161 (28.65)	5383 (71.35)	7448 (98.73)	96 (1.27)	6846 (90.75)	698 (9.25)	5447 (72.20)	2097 (27.80)
Others	49 (28.16)	125 (71.84)	170 (97.70)	4 (2.30)	145 (83.33)	29 (16.67)	143 (82.18)	31 (17.82)
Wealth index								
Poorest	365 (18.85)	1571 (81.15)	1900 (98.14)	36 (1.86)	1714 (88.53	222 (11.47)	1383 (71.44)	553 (28.56)
Middle	440 (30.24)	1015 (69.76)	1435 (98.63)	20 (1.37)	1325 (91.07)	130 (8.93)	1055 (72.51)	400 (27.49)
Richest	544 (43.69)	701 (56.31)	1239 (99.52)	6 (0.48)	1156 (92.85)	89 (7.15)	963 (77.35)	282 (22.65)
Place of residence								
Urban	1713 (27.22)	4580 (72.78)	6210 (98.68)	83 (1.32)	5701 (90.59)	592 (9.41)	4540 (72.14)	1753 (27.86)
Rural	497 (34.87)	928 (65.12)	1408 (98.81)	17 (1.19)	1290 (90.53)	135 (9.47)	1050 (73.68)	375 (26.32)
Division								
Barisal	225 (32.70)	463 (67.30)	685 (99.56)	3 (0.44)	615 (89.39)	73 (10.61)	449 (65.26)	239 (34.74)
Chattogram	472 (30.16)	1093 (69.84)	1546 (98.79)	19 (1.21)	1385 (88.50)	180 (11.50)	1152 (73.61)	413 (26.39)
Dhaka	479 (31.16)	1058 (68.84)	1521 (98.96)	16 (1.04)	1413 (91.93)	124 (8.07)	1272 (82.76)	265 (17.24)
Khulna	292 (27.01)	789 (72.99)	1074 (99.35)	7 (0.65)	999 (92.41)	82 (7.59)	690 (63.83)	391 (36.17)
Mymensingh	143 (30.36)	328 (69.64)	454 (96.39)	17 (3.61)	419 (88.96)	52 (11.04)	297 (63.06)	174 (36.94)
Rajshahi	215 (24.38)	667 (75.62)	871 (98.75)	11 (1.25)	817 (92.63)	65 (7.37)	617 (69.95)	265 (30.05)
Rangpur	242 (27.19)	648 (72.81)	866 (97.30)	24 (2.70)	822 (92.36)	68 (7.64)	721 (81.01)	169 (18.99)
Sylhet	142 (23.51)	462 (76.49)	601 (99.50)	3 (0.50)	521 (86.26)	83 (13.74)	392 (64.90)	212 (35.10)
Religion								
Islam	1898 (28.67)	4723 (71.33)	6536 (98.72)	85 (1.28)	6012 (90.80)	609 (9.20)	4782 (72.22)	1839 (27.78)
Others	312 (28.44)	785 (71.56)	1082 (98.63)	15 (1.37)	979 (89.24)	118 (10.76)	808 (73.66)	289 (26.34)
Child Education book at home								
Yes	1644 (40.83)	2382 (89.17)	3991 (99.13)	35 (0.87)	3778 (93.84)	248 (6.16)	2927 (72.70)	1099 (27.30)
No	566 (15.33)	3126 (84.67)	3627 (98.24)	65 (1.76)	3213 (87.03)	479 (12.97)	2663 (72.13)	1029 (27.87)
Age of child (in years)								
3	638 (16.29)	3279 (83.71)	3854 (98.39)	63 (1.61)	3481 (88.87)	436 (11.13)	2744 (70.05)	1173 (29.95)
4	1572 (41.36)	2229 (58.64)	3764 (99.03)	37 (0.97)	3510 (92.34)	291 (7.66)	2846 (74.88)	955 (25.12)
Child's sex								
Male	1086 (27.33)	2887 (72.67)	3922 (98.72)	51 (1.28)	3572 (89.91)	401 (10.09)	2740 (68.97)	1233 (31.03)
Female	1124 (30.01)	2621 (69.99)	3696 (98.69)	49 (1.31)	3419 (91.30)	326 (8.70)	2580 (74.24)	895 (25.76)
Stunted								
Yes	411 (18.80)	1775 (81.20)	2155 (98.58)	31 (1.42)	1933 (88.43)	253 (11.57)	1551 (70.95)	635 (29.05)
No	1799 (32.52)	3733 (67.48)	5463 (98.75)	69 (1.25)	5058 (91.43)	474 (8.57)	4039 (73.01)	1493 (26.99)
Wasted								
Yes	221 (29.04)	540 (70.96)	755 (99.21)	6 (0.79)	692 (90.93)	69 (9.07)	552 (72.54)	209 (27.46)
No	1989 (28.59)	4968 (71.41)	6863 (98.65)	94 (1.35)	6299 (90.54)	658 (9.46)	5038 (72.42)	1919 (27.58)
Underweight								
Yes	404 (21.15)	1506 (78.85)	1891 (99.01)	19 (0.99)	1706 (89.32)	204 (10.68)	1373 (71.88)	537 (28.12)
No	1806 (31.10)	4002 (68.90)	5727 (98.61)	81 (1.39)	5285 (91.00)	523 (9.00)	4217 (72.61)	1591 (27.39)
Overweight								
Yes	46 (41.07)	66 (58.93)	107 (95.54)	5 (4.46)	99 (88.39)	13 (11.61)	83 (74.11)	29 (25.89)
No	2164 (28.45)	5442 (71.55)	7511 (98.75)	95 (1.25)	6892 (90.61)	714 (9.39)	5507 (72.40)	2099 (57.60)
Sanitation								
Yes	2144 (28.70)	5326 (71.30)	7373 (98.70)	97 (1.30)	6767 (90.59)	703 (9.41)	5397 (72.25)	2073 (27.75)
No	66 (26.61)	182 (73.39)	245 (98.79)	3 (1.21)	224 (90.32)	24 (9.68)	193 (77.51)	56 (22.49)
Early childhood education programs								
Yes	861 (58.53)	610 (41.47)	1455 (98.91)	16 (1.09)	1384 (94.09)	87 (5.91)	1080 (73.42)	391 (26.58)
No	1349 (21.59)	4898 (78.41)	6163 (98.66)	84 (1.34)	5607 (89.76)	640 (10.24)	4510 (72.19)	1737 (27.81)
Inadequate supervision								
Yes	159 (22.08)	561 (77.92)	704 (97.78)	16 (2.22)	629 (87.36)	91 (12.64)	521 (72.36)	199 (27.64)
No	2051 (29.31)	4947 (70.69)	6914 (98.80)	84 (1.20)	6362 (90.91)	636 (9.09)	5069 (72.43)	1929 (27.57)
Toys								
Yes	1812 (28.41)	4566 (71.59)	6303 (98.82)	75 (1.18)	5844 (91.63)	534 (8.37)	4492 (70.43)	1886 (29.57)
No	398 (29.70)	942 (70.30)	1315 (98.13)	25 (1.87)	1147 (85.60)	193 (14.40)	1098 (81.94)	242 (18.06)
Mass media								
Yes	1327 (28.34)	3356 (71.66)	4627 (98.80)	56 (1.20)	4216 (90.03)	467 (9.97)	3401 (72.62)	1282 (27.38)
No	883 (29.09)	2152 (70.91)	2991 (98.55)	44 (1.45)	2775 (91.43)	260 (8.57)	2189 (72.13)	846 (27.87)
Mother's education								
Primary incomplete	150 (14.62)	876 (85.38)	1010 (98.44)	16 (1.56)	905 (88.21)	121 (11.79)	795 (45.50)	258 (24.50)
Primary complete	387 (20.55)	1496 (79.45)	1851 (98.3)	32 (1.7)	1686 (89.54)	197 (10.46)	742 (72.32)	284 (27.68)
Secondary incomplete	1190 (31.68)	2566 (68.32)	3721 (99.07)	35 (0.93)	3427 (91.24)	329 (8.76)	1345 (71.43)	538 (28.57)
Secondary complete or higher	483 (45.87)	570 (54.13)	1036 (98.39)	17 (1.61)	973 (92.40)	80 (7.60)	2708 (72.10)	1048 (27.90)

Instead of conducting a hypothesis‐driven inferential analysis, our study was intended to be a predictive modeling study. The nonlinear, imbalanced, and high‐dimensional nature of the MICS data set, which violates assumptions of many parametric statistical tests, made this methodological decision appropriate. As a result, we did not apply traditional significance tests such as t‐tests or regression coefficients. Rather, we used a variety of metrics, including accuracy, precision, recall, F1‐score, specificity, and AUC‐ROC, which offer more reliable indicators of generalizability than *p*‐values, to assess supervised machine learning models considering the nature of the data. To measure the relative relevance of indicators, we also used feature importance analysis, which provides deeper insights than separate significance tests. Although statistical testing can be a useful addition to future research, early risk identification and prediction were the main emphasis of this study.

The performance of five supervised machine learning models—CART, Random Forest (RF), XGBoost, Logistic Regression (LR), and Support Vector Machines (SVM)—was evaluated across four developmental domains: literacy‐numeracy, physical, learning, and social‐emotional. With an accuracy of 0.69, precision of 0.47, recall of 0.63, and F1‐score of 0.54, CART performed the worst in the literacy‐numeracy domain. Additionally, its specificity was 0.63 and its AUC was 0.72. With an accuracy of 0.73, precision of 0.53, recall of 0.66, and F1‐score of 0.59, Random Forest (RF), on the other hand, performed best in this domain. Additionally, RF showed a more robust and balanced performance with an AUC of 0.78 and specificity of 0.66. With an accuracy of 0.78, precision of 0.99, recall of 0.78, F1‐score of 0.87, AUC of 0.56, and specificity of 0.78, Support Vector Machines (SVM) received the lowest scores in the physical development domain. However, Random Forest (RF) performed the best, achieving remarkable results across the board with an accuracy of 0.97, precision of 0.99, recall of 0.99, F1‐score of 0.99, an AUC of 0.62, and specificity of 0.95. With an accuracy of 0.65, precision of 0.92, recall of 0.67, F1‐score of 0.78, AUC of 0.60, and specificity of 0.67, Logistic Regression had the lowest performance for learning development. As before, Random Forest (RF) performed well and consistently across all evaluation metrics, leading with the highest scores: accuracy of 0.83, precision of 0.91, recall of 0.90, F1‐score of 0.90, AUC of 0.62, and specificity of 0.89. With an accuracy of 0.60, precision of 0.75, recall of 0.65, F1‐score of 0.69, AUC of 0.55, and specificity of 0.65, CART performed the worst in the social‐emotional development domain. With an accuracy of 0.64, precision of 0.74, recall of 0.77, F1‐score of 0.75, AUC of 0.60, and specificity of 0.77, Random Forest (RF) once again outperformed other models in this domain (Table 2).

**Table 2:** Performance of all machine learning models across four domains by accuracy, precision, recall, F1‐Score, AUC‐ROC score, and specificity.

Models	Domain	Accuracy	Precision	Recall	F1‐score	AUC ROC score	Specificity
CART	Literacy‐numeracy	0.69	0.47	0.63	0.54	0.72	0.63
	Physical	0.95	0.99	0.96	0.98	0.58	0.95
	Learning	0.76	0.91	0.82	0.86	0.55	0.81
	Social‐emotional	0.60	0.75	0.65	0.69	0.55	0.65
Random forest	Literacy‐numeracy	0.73	0.53	0.66	0.59	0.78	0.66
	Physical	0.97	0.99	0.99	0.99	0.62	0.95
	Learning	0.83	0.91	0.90	0.90	0.62	0.89
	Social‐emotional	0.64	0.74	0.77	0.75	0.60	0.77
XGBoost	Literacy‐numeracy	0.73	0.52	0.65	0.58	0.77	0.65
	Physical	0.96	0.99	0.98	0.99	0.61	0.93
	Learning	0.82	0.91	0.89	0.90	0.60	0.88
	Social‐emotional	0.63	0.75	0.73	0.74	0.60	0.72
Logistic regression	Literacy‐numeracy	0.72	0.51	0.70	0.59	0.77	0.70
	Physical	0.78	0.99	0.79	0.88	0.55	0.79
	Learning	0.65	0.92	0.67	0.78	0.60	0.67
	Social‐emotional	0.65	0.76	0.55	0.64	0.56	0.55
Support vector machines	Literacy‐numeracy	0.70	0.48	0.70	0.59	0.78	0.69
	Physical	0.78	0.99	0.78	0.87	0.56	0.78
	Learning	0.64	0.93	0.66	0.77	0.60	0.66
	Social‐emotional	0.62	0.59	0.58	0.58	0.57	0.45

**Figure 3:**
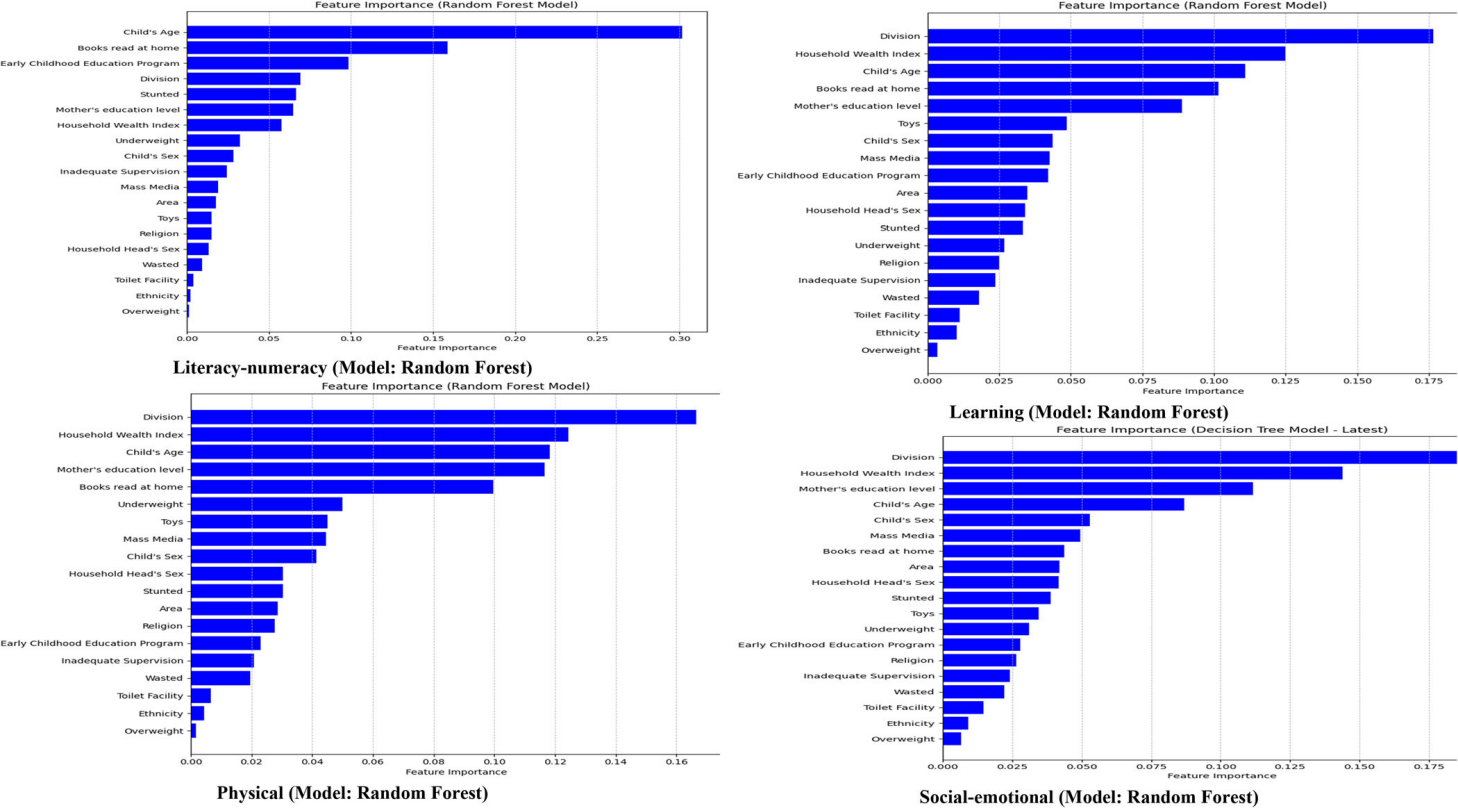
Random Forest's Feature Importance for all four domains. Literacy‐numeracy (Model: Random Forest). Physical (Model: Random Forest). Learning (Model: Random Forest). Social‐emotional (Model: Random Forest).

In the Random Forest models for various developmental domains, the key predictors of outcomes vary, but some common themes emerge. For literacy‐numeracy, the most significant predictors are Child's Age and Books Read at Home, highlighting the importance of early learning experiences and age‐related development. Other important factors include participation in an Early Childhood Education Program, Division (likely reflecting regional or geographic influences), Stunted (indicating physical health), and the Mother's Education Level, all contributing to literacy and numeracy outcomes. In physical development, the most influential factors are Division, Household Wealth Index, and Child's Age, emphasizing the role of socioeconomic status and age. Additional important features include Mother's Education Level, Books Read at Home, and Underweight, underscoring the impact of parental education, early learning, and nutrition on physical health. For learning development, Division, Household Wealth Index, and Child's Age are the key drivers, reflecting the influence of socioeconomic conditions and developmental stages. The Books Read at Home and the Mother's Education Level are also critical, pointing to the importance of parental involvement and early educational environments. In social‐emotional development, the most significant factors are Division, Household Wealth Index, and Mother's Education Level, which strongly affect emotional development. Additionally, Child's Age and Child's Sex play important roles, illustrating how age and gender influence emotional growth. Overall, across all domains, socioeconomic status, regional factors, and maternal education emerge as the key drivers influencing a child's development (Figure 3).

## 4 Discussion

We have used MICS6 (2019) data to study children's overall development in the domains of literacy‐numeracy, physical, learning, and social‐emotional development. This study illustrates the effective application of supervised machine learning (ML) techniques in predicting early childhood developmental outcomes using data from the [12] survey. By evaluating five classifiers—CART, Random Forest, XGBoost, Logistic Regression, and Support Vector Machines (SVM)—across four developmental domains, we demonstrate the strengths and limitations of each model in a health and developmental context. Among all models, Random Forest (RF) consistently outperformed the others in all domains. RF achieved the highest scores in accuracy, precision, recall, F1 score, and AUC‐ROC, confirming its robustness in handling structured and moderately high‐dimensional survey data. These findings are consistent with previous studies demonstrating RF's strong predictive capabilities in health and behavioral datasets [42, 43].

Compared to other supervised machine learning algorithms across multiple domains, Random Forest performed better than all other models. Within the domains of Learning Development, Physical Development, Social‐Emotional Development, and Literacy and Numeracy, RF outperformed in terms of all major evaluation measures, including F1 score, AUC‐ROC, accuracy, precision, and recall. We also analyzed the importance of features within each domain to identify the most influential variables contributing to model predictions. Feature importance provides insight into the contribution of each input variable to the model's output, helping to interpret model behavior. In this context, estimating the importance of features aids not only in understanding model inference but also in forming hypotheses about potential causal relationships between child, family, and environmental factors and developmental outcomes. This approach supports the use of the F1 score as an interpretable performance metric that balances precision and recall, which is particularly important when dealing with imbalanced health data [44].

Important factors in the literacy‐numeracy domain, considering feature importance are the child's age, the number of books read at home, division, the mother's educational level, the wealth index of the family, as well as participation in early childhood education programs. These data imply that early educational experiences and socioeconomic circumstances are important for a child's development of literacy and numeracy. According to Hossain et al., early literacy and numeracy abilities are less likely to develop in comparison to peers in 36–47‐month‐old males, moderately stunted children, children of mothers with just a primary education or no education, and children from the poorest to the wealthiest homes [45]. The availability of children's books at home and the children's literacy and numeracy index (LNI) are significantly correlated. It indicates that in nations where a larger number of families own at least one book for children, a more significant share of kids is progressing well in their language, numeracy, and literacy abilities [46]. A study suggested that parental education has a positive impact on child literacy and numeracy [47].

In terms of physical development, the most significant factors were found to be the nutritional indicators like being underweight or overweight, availability of sanitary facilities, child's age, the mother's educational attainment, number of books read at home, wealth index, and participation in early childhood education programs. This suggests that several factors related to health, parental education, and socioeconomic status all have a substantial impact on physical development. A similar study conducted by Rahman et al. also found that the ECD status was significantly associated with the child's age and sex, the mother's level of education, and the family wealth index [10]. Ashfikur et al. found that children from poor to the poorest family background have at least one form of malnutrition [48].

According to Alam et al., in Bangladesh, the prevalence of early childhood development varies significantly throughout the divisions. Most divisions are falling behind in terms of ECD when compared to the Dhaka division. However, kids in the Mymensingh division are more susceptible to underdeveloped physical and social‐emotional skills [49]. The wealth index is a significant factor in a child's physical health. This is in line with previous studies showing that physical factors are greatly influenced by the socioeconomic position of the household [50]. Children from Bangladeshi households with the lowest incomes had 38% higher probabilities of malnutrition than children from the wealthiest families [51]. Research conducted by Stamenkovic et al. in Serbia found that the most significant factor influencing a child's nutritional status was found to be the mother's level of education [52].

The most important variables around learning development are the number of books read at home, the mother's educational attainment, the wealth index, and the involvement in early childhood education programs. According to these results, learning development greatly benefits from a stable family environment that is blessed with educational resources and financial stability. A child's ability to read well at a young age is strongly connected to the overall educational atmosphere within their home. This involves more than just having books for them to read; it also involves having access to various educational tools and resources that can help enhance their intellectual growth [53]. According to Einglund et al., more educated mothers are better able to assist their preschool‐aged children in addressing problems [54]. The socioeconomic status of parents has an association with the cognitive abilities of their children [55].

Finally, the mother's educational attainment, the child's age, the sex of the household head, the child's ethnicity, and the wealth index are important variables in the social‐emotional development domain. These findings emphasize how a child's social‐emotional development is influenced by both socioeconomic status and demographic characteristics. Maternal involvement and kids' cognitive or social‐emotional development are not significantly correlated. Children who have two or more different kinds of toys showed a positive relationship with their social and emotional development [56].

Our feature importance findings demonstrate that maternal education, household socioeconomic status, regional differences, and the setting for learning at home all have a significant impact on child development. Therefore, policies should extend parental literacy activities, give priority to equity‐focused early childhood programs in underprivileged areas, and boost access to free or inexpensive children's books. The importance of child nutrition—underweight, stunting, and wasting—makes it imperative that early education strategies incorporate nutritional support [57]. Lastly, in situations where resources are scarce, inexpensive activities like play, singing, and storytelling can effectively stimulate [13]. These treatments can be directed at the children who are most at risk, thanks to predictive modeling, which guarantees more fair results and effective use of resources.

### 4.1 Strengths and Limitations

To the best of our knowledge, this is the first study to use five supervised machine learning models to introduce predictive modeling across four domains of children's well‐being. We seek to offer a holistic study that encompasses literacy, numeracy, physical, learning, and social‐emotional domains by utilizing a comprehensive methodology. Using the Synthetic Minority Over‐sampling Technique (SMOTE) to address the problem of data imbalance is a crucial component of our research. Empirical evidence has demonstrated that SMOTE improves model performance, especially in situations when there is an uneven distribution of classes. By utilizing this method, we make sure that our prediction models are more resilient, dependable, and able to recognize patterns and trends in the data, providing a more accurate evaluation of children's well‐being in these fundamental developmental domains. Our study has several drawbacks despite its strengths. We were reliant on secondary data, which meant we did not influence the choice of variables, the data quality, or the measurement indicators used. Furthermore, the data on child development in the study only covered children between the ages of three and four, which made it challenging to compare younger children's developmental growth to that of 3‐ and 4‐year‐olds. Extra information ranging from birth to 5 years is essential for achieving a deeper understanding of children's development on a national scale.

## 5 Conclusion

Our analysis revealed unique characteristics influencing each developmental domain. The child's age, the number of books available at home, the mother's education, the financial status of the household, and involvement in early education programs all had an impact on the literacy‐numeracy results. Socioeconomic conditions, sanitation, and nutritional status were all strongly correlated with physical development. While socio‐emotional development was linked to age, maternal education, household gender dynamics, and wealth, learning development was influenced by the home learning environment, maternal education, and family wealth. These results emphasize how important early involvement, nutrition, parental education, and socioeconomic resources are in promoting a child's development. Policymakers and practitioners can create immediate, focused interventions to lessen developmental inequities by targeting these issues. Future studies should confirm these findings and create age‐appropriate, culturally aware instruments to better record developmental paths.

## Author Contributions

**Faizul Islam:** data curation, formal analysis, investigation, methodology, project administration, validation, writing – original draft. **Golam Morshed Suhel:** data curation, formal analysis, investigation, methodology, writing – original draft. **Mahmud Afroz:** data curation, formal analysis, methodology, writing – review and editing. **Md. Aminul I. Apu:** data curation, formal analysis, methodology, writing – review and editing. **Md. Jewel Rana:** data curation, methodology, project administration, writing – review and editing. **Tofajjel Hossain:** data curation, methodology, project administration, writing – review and editing. **Zaibunnesa Ziba:** data curation, methodology, project administration, writing – review and editing. **Md Fahim Shariar:** data curation, methodology, project administration, writing – review and editing. **Mohammad Nayeem Hasan:** data curation, methodology, project administration, validation, writing – review, and editing.

## Conflicts of Interest

The authors declare no conflicts of interest.

## Transparency Statement

The lead author Mohammad Nayeem Hasan affirms that this manuscript is an honest, accurate, and transparent account of the study being reported; that no important aspects of the study have been omitted; and that any discrepancies from the study as planned (and, if relevant, registered) have been explained.

## Data Availability

The data sets were accessible to the public free of charge (https://mics.unicef.org/surveys). All analyses were conducted using the Bangladesh MICS 2019 data set. This study did not create or analyze any new data, and the data are freely available.
